# What drives mortality among HIV patients in a conflict setting? A prospective cohort study in the Central African Republic

**DOI:** 10.1186/s13031-019-0236-7

**Published:** 2019-11-14

**Authors:** Thomas Crellen, Charles Ssonko, Turid Piening, Marcel Mbeko Simaleko, Karen Geiger, M. Ruby Siddiqui

**Affiliations:** 1 Médecins Sans Frontières Hollande, Avenue Barthelemy Boganda, PK4, Bangui, BP 1793 Central African Republic; 2 Mahidol-Oxford Tropical Medicine Research Unit, 420/6 Rajvithi Road, Tungphyathai, Bangkok, 10400 Thailand; 3 Médecins Sans Frontières, The Manson Unit, Chancery Exchange, 10 Furnival Street, London, EC4A 1AB UK; 4 Médecins Sans Frontières, Am Köllnischen Park 1, 10179 Berlin, Germany; 5 Ministre de la Santé Publique et de la Population, Bangui, BP 883 Central African Republic

**Keywords:** Antiretroviral therapy, Conflict setting, Survival analysis, Cohort study, Central African Republic

## Abstract

**Background:**

Provision of antiretroviral therapy (ART) during conflict settings is rarely attempted and little is known about the expected patterns of mortality. The Central African Republic (CAR) continues to have a low coverage of ART despite an estimated 110,000 people living with HIV and 5000 AIDS-related deaths in 2018. We present results from a cohort in Zemio, Haut-Mboumou prefecture. This region had the highest prevalence of HIV nationally (14.8% in a 2010 survey), and was subject to repeated attacks by armed groups on civilians during the observed period.

**Methods:**

Conflict from armed groups can impact cohort mortality rates i) directly if HIV patients are victims of armed conflict, or ii) indirectly if population displacement or fear of movement reduces access to ART. Using monthly counts of civilian deaths, injuries and abductions, we estimated the impact of the conflict on patient mortality. We also determined patient-level risk factors for mortality and how the risk of mortality varies with time spent in the cohort. Model-fitting was performed in a Bayesian framework, using logistic regression with terms accounting for temporal autocorrelation.

**Results:**

Patients were recruited and observed in the HIV treatment program from October 2011 to May 2017. Overall 1631 patients were enrolled and 1628 were included in the analysis giving 48,430 person-months at risk and 145 deaths. The crude mortality rate after 12 months was 0.92 (95% CI 0.90, 0.93). Our model showed that patient mortality did not increase during periods of heightened conflict; the odds ratios (OR) 95% credible interval (CrI) for i) civilian fatalities and injuries, and ii) civilian abductions on patient mortality both spanned unity. The risk of mortality for individual patients was highest in the second month after entering the cohort, and declined seven-fold over the first 12 months. Male sex was associated with a higher mortality (odds ratio 1.70 [95% CrI 1.20, 2.33]) along with the severity of opportunistic infections (OIs) at baseline (OR 2.52; 95% CrI 2.01, 3.23 for stage 2 OIs compared with stage 1).

**Conclusions:**

Our results show that chronic conflict did not appear to adversely affect rates of mortality in this cohort, and that mortality was driven predominantly by patient-specific risk factors. The risk of mortality and recovery of CD4 T-cell counts observed in this conflict setting are comparable to those in stable resource poor settings, suggesting that conflict should not be a barrier in access to ART.

## Introduction

Sub-Saharan Africa (SSA) exhibits disproportionate levels of morbidity and mortality from both infectious diseases and conflict. Despite SSA accounting for 14.2% of the world’s population [1], estimates by UNAIDS for 2018 gave the numbers of people living with HIV (PLHIV) in SSA at 25.6 million and AIDS-related deaths at 470,000; representing 67.5 and 61.0% respectively of the global totals [2]. The Uppsala Conflict Data Program reported 20 separate conflicts in SSA in 2018; the African region had the highest number of fatalities from non-state conflicts (5408; 45.7%) and attacks against civilians (2888; 70.0%) [3]. This is consistent with the disproportionate levels of violent conflict in SSA since 2010 [4, 5]. Examples of regions in SSA with a dual burden of HIV and conflict in 2018 include South Sudan, which had a national adult prevalence of 2.5% in 2018 [2] though higher estimates (3.1–6.8%) in conflict affected southern states [6], the Cabo Delgado province of northern Mozambique which was subject to attacks by Islamic extremists in 2018 and had a HIV prevalence of 12.6% in the same year [2], and Maniema province of the eastern Democratic Republic of Congo, which saw clashes between government forces and rebel groups in 2018, had an estimated HIV prevalence of 3.4% compared with a national average of 1.0% [7].

Nations in SSA affected by conflict have been shown to have worse health outcomes, such as rates of mortality for mothers and children under-five [8, 9]. The relationship between conflict and the transmission of infectious disease is more complex and contested in the literature. Conflict in SSA between 1997 and 2010 did not appear to result in higher transmission rates for falciparum malaria and the pre-conflict trend was generally maintained [10]. For HIV, a number of multi-site studies in SSA have argued there is no evidence that infection incidence increases during conflict when compared with pre-conflict rates [11–13]. However, a cohort study of police officers in Guinea-Bissau during the 1998–9 civil war reached the opposite conclusion for the incidence of HIV-1 [14], and the observations of stable or decreasing HIV incidence before and during conflict from cross-sectional surveys could be confounded by higher rates of, unobserved, AIDS-related mortality [15].

Provision of anti-retroviral therapy (ART) during conflict has, historically, not been prioritised due to a perception that this was too difficult to achieve [16]. In the face of long term conflicts in areas with a high HIV burden, there was concern in the humanitarian community that “treatment cannot wait” [13, 17]. In 2010 the medical organisation Médecins Sans Frontières (MSF) reported results from 22 ART programs in conflict or post-conflict settings in SSA, finding that patient outcomes were comparable to those in stable resource-limited settings [18]. These findings were also shown in a systematic review and meta-analysis that found mortality and loss to follow-up (LTFU) of patients on ART in conflict settings after 12 months was 9.0 and 8.1% respectively, which are within the range of mortality and LTFU estimates from non-conflict settings [19].

The Central African Republic (CAR) is one of the world’s least developed nations [20] and faces a number of challenges in healthcare provision including disruption of services due to instability and civil conflict along with a lack of infrastructure, particularly outside of the capital Bangui. At the onset of the study taking place, CAR had an estimated 120,000 PLHIV and 11,000 AIDS-related deaths in 2013 [21] giving a mortality rate of 91 deaths per 1000 PLHIV annually, which was the highest in the world [22]. More recent estimates by UNAIDS for 2018 suggest there are 110,000 PLHIV in CAR (uncertainty interval [UI] 90,000 – 140,000) and 4800 AIDS-related deaths (UI 3700 – 6400) [2]. In 2018, it was estimated that 36% (UI 30–45%) of people living with HIV were receiving ART [2].

MSF conducted a nationwide HIV seroprevalence study in 2010 which found the Haut-Mbomou prefecture to have the highest prevalence in CAR; 14.8% compared with a prevalence of 5.9% nationally among individuals 15–49 years of age [23]. Access to treatment in CAR was particularly low at this time, 13.8% in 2010 [21]. The health centre in Zemio, Haut-Mboumou prefecture has been supported by MSF since 2010 and an ART cohort was established in October 2011. This was initially a response to an influx of Congolese refugees and internally displaced persons (IDPs), who had fled their homes due to repeated attacks by the Lord’s Resistance Army (LRA) during 2008–10. The region has since faced repeated violent incursions from armed groups [24]. The Zemio HIV treatment program therefore represented an attempt to provide access to ART for a population with a high disease burden in a conflict setting.

Reports of HIV patient outcomes generally focus on summary statistics after defined intervals (6 or 12 months) and are rarely explored as a detailed time series. It is important to consider temporal variation in patient mortality as this has implications for management of patient care and allocation of resources during ART programs. For instance, the risk of mortality among patients in sub-Saharan Africa is particularly high in the first 3 months of ART provision [25, 26] due to individuals seeking treatment at a later stage of disease progression [27]. In the case of studies on the impact of conflict, longitudinal data is particularly important as instability is rarely temporally uniform and often consists of periods of relative calm punctuated by acute violence [17]. Conflict from armed groups can impact mortality rates i) directly if HIV patients are victims of armed conflict, or ii) indirectly if population displacement or fear of movement reduces adherence to ART [28].

In this study, we address both temporal variation in survival rates and the impact of conflict on patient mortality. We use a logistic regression survival analysis where the underlying rate of mortality is permitted to vary by month. The impact of the conflict is measured though aggregated monthly counts of civilian fatalities, injuries and abductions from armed groups. We determine associations between the intensity of conflict with the risk of monthly mortality, in addition to exploring the effect of patient-level risk factors.

## Methods

This research fulfilled the exemption criteria set by the MSF Ethical Review Board (ERB) for a posteriori analyses of routinely collected clinical data and thus did not require MSF ERB review. This study was conducted with permission from the Medical Director Sidney Wong (MSF-Operational Centre Amsterdam). Patients in the Zemio HIV cohort gave written consent for anonymised data to be used for research purposes.

**Fig. 1:**
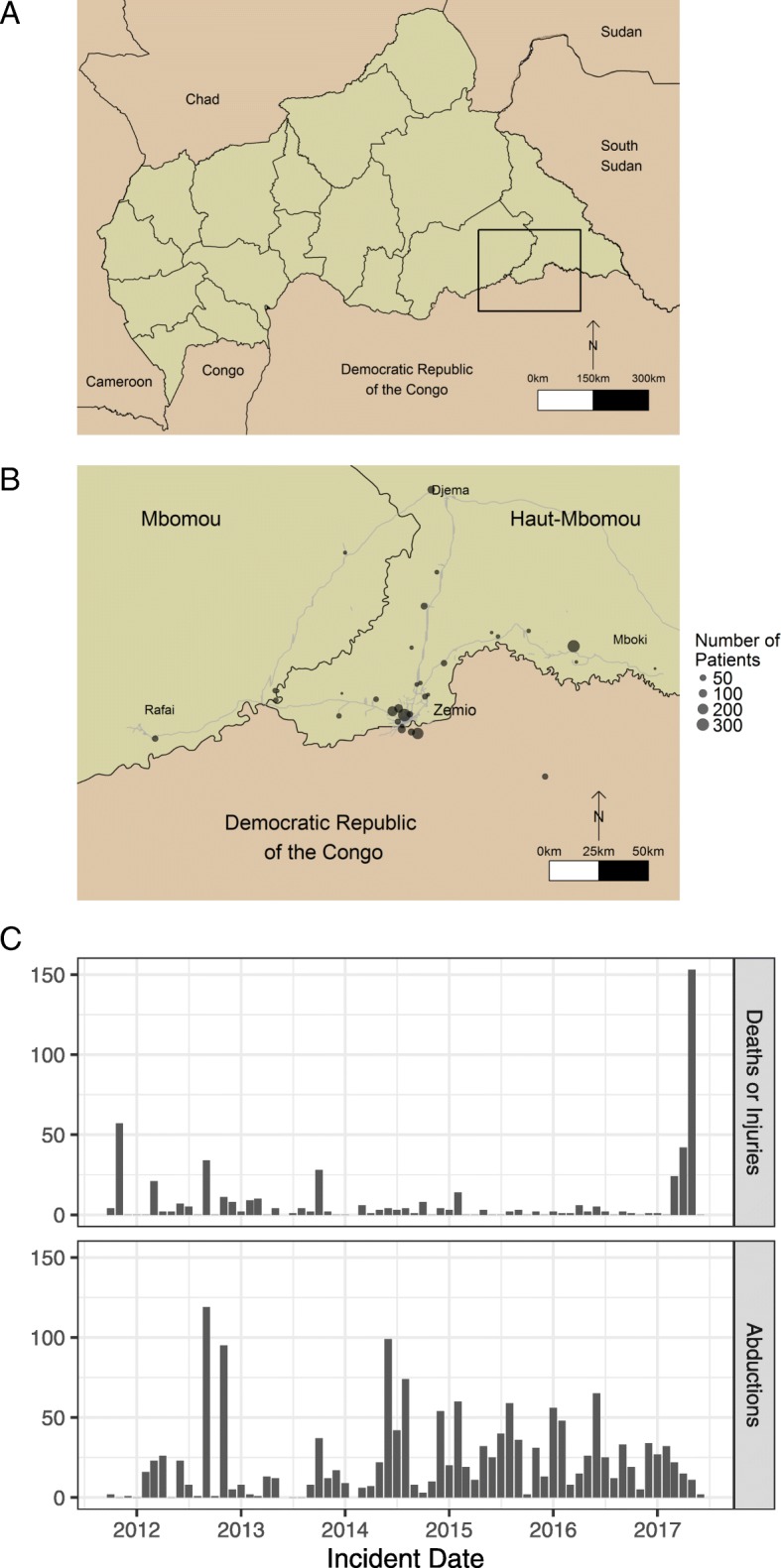
The Central African Republic (CAR) is shown in a regional context (**a**), with the country divided by prefectures. The study area is indicated by the box in the south east of CAR. The town of Zemio is shown in context (**b**), within Haut-Mbomou prefecture and adjacent to Mbomou prefecture. Nearby towns where patients were drawn from for the HIV treatment program (Rafai, Djema and Mboki) and the Bas-Uele province of the Democratic Republic of the Congo are also shown. The majority of patients were drawn from around the town of Zemio, including internally displaced person and refugee camps resulting from conflict in 2010 by the Lord’s Resistance Army. Grey lines indicate the road network in the CAR. The number of patients recruited into the cohort from each location is indicated by the size of the points (*n* = 1631). A time series of civilian deaths / injuries and abductions by armed groups in the patient catchment area details the evolution of the conflict in this region from October 2011 – May 2017 (**c**)

**Fig. 2:**
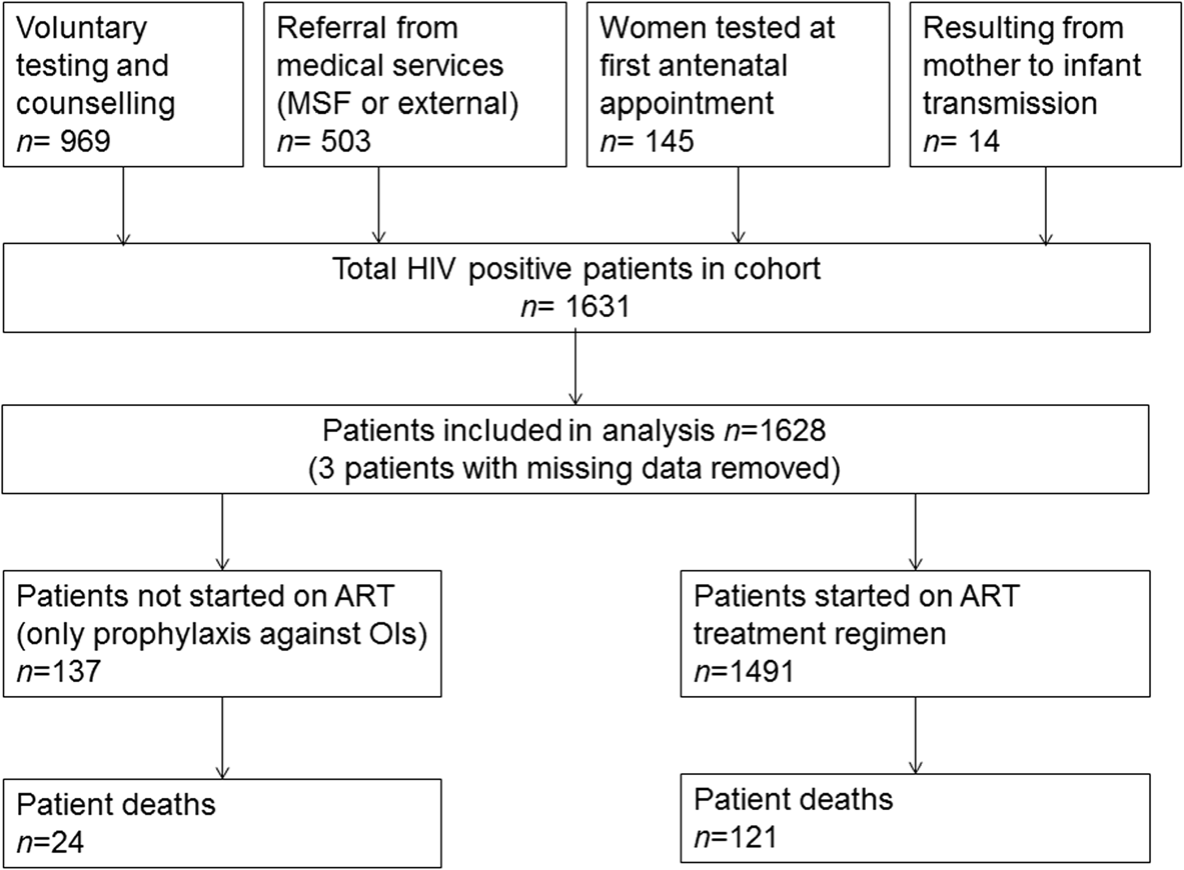
Flow chart for patient recruitment into the Zemio HIV cohort, Central African Republic between October 2011 – May 2017 and inclusion into the statistical analysis. The figures for patients started on ART / not started on ART and deaths are from those included in the statistical analysis

We used data from the treatment program’s commencement on 18th October 2011 until 31st May 2017. Patients were recruited prospectively throughout the study period and were drawn from the surrounding area, which includes the Bas-Uele province of neighbouring Democratic Republic of Congo along with Congolese refugee camps and IDP camps around Zemio (Fig. 1). The target population consisted mainly of the local Azande people who work predominantly as subsistence farmers (Table 1), and Fulani pastoralists who also reside in the region. Patients qualified for inclusion in the study i) if they volunteered for HIV testing and counselling at the Zemio health centre (usually following contact with community health workers), ii) if they were referred to the testing and counselling program after an appointment in Zemio or nearby health facilities, or iii) during the first antenatal visit for pregnant women. Infants testing positive for HIV after birth were also enrolled into the study (Fig. 2). The study used data from medical records collected during patient enrolment and consultations, which were entered into a modified Microsoft Access database (FUCHIA) used by MSF for monitoring of HIV cohorts.

**Table 1:** Characteristics of the 1631 HIV-positive patients enrolled in the Zemio HIV cohort, Central African Republic from October 2011–May 2017. Interquartile range = IQR, ART = antiretroviral therapy, OIs = opportunistic infections. Loss to follow up is defined as patients not present for an appointment > 12 months before the end of the program

Total patients enrolled in the Zemio HIV cohort	1631 (4107 person-years at risk)
Patients enrolled on ART	1491/1631 (91.4%)
Female patients	1147/1631 (70.3%)
Median age at first appointment (IQR)	29 years (22, 37)
Deaths	148
Loss to follow up	183
CD4 cell count/μl, baseline (IQR), *n* = 1017	268 (148, 427)
CD4 cell count/μl, 6 months (IQR), *n* = 442	403 (240, 634)
CD4 cell count/μl, 12 months (IQR), *n* = 321	433 (264, 636)
Clinical Stage 1 OIs at baseline	241/1628 (14.8%)
Clinical Stage 2 OIs at baseline	403/1628 (24.7%)
Clinical Stage 3 OIs at baseline	789/1628 (48.5%)
Clinical Stage 4 OIs at baseline	195/1628 (12.0%)
Occupation: Infants/ Pupil / Student	148/1628 (9.1%)
Occupation: Farmers / Fishermen / Domestic	1272/1628 (78.1%)
Occupation: Small commerce	139/1628 (8.5%)
Occupation: Civil servant/ official	26/1628 (1.6%)
Occupation: Other /not recorded	43/1628 (2.6%)

Patients were tested for HIV with the Determine™ HIV-1/2 (Alere) antigen/antibody test and Uni-Gold™ Recombigen® (Trinity Biotech) antibody test using whole blood; a positive diagnosis from both tests was required before diagnosing a patient as positive and the tests were repeated in the case of discordant results. Infants were tested with PCR every 6 months up to 18 months after birth either i) from a dry blood spot sent to Global Clinical and Viral Laboratory, South Africa prior to 2017 or ii) using Xpert® HIV-1 Qual (Cepheid) from whole blood on-site in 2017.

The Zemio program offered anti-retrovirals (ARVs) to individuals based on WHO guidelines [29–31]; from 2011 to 2012 for patients with CD4 < 350 cells/μL, from 2013 to 2016 with < 500 cells/μL and for all patients regardless of CD4 count from 2017 (universal test and treat). First line therapy was typically tenofovir/lamivudine/ efavirenz (TDF/3TC/EFV) combination therapy, second line therapy was zidovudine/lamivudine/ lopinavir/ritonavir (AZT/3TC/LPVr). Patients aged ≤15 years old were treated with paediatric drug formulations. Patients with CD4 counts above the threshold were given prophylactic treatment against opportunistic infections (OIs) and retested every 6 months until they were eligible to initiate. Co-trimoxazole prophylaxis was given to all patients before the initiation of ART and continued during ART. Patients were given appointments at least every 3 months when ART was provided and clinical signs of OIs were recorded and treated. All diagnostics, treatment and appointments were provided to patients free of charge by MSF.

The Zemio HIV program was mainly nurse driven, an expatriate nurse was present to provide training and guidance to two national staff nurse consultants and *secouristes* (trained lay workers) who provided HIV testing and counseling and psychosocial care to patients. HIV test results were confirmed by a lab technician supported by *secouristes*. A medical doctor was consulted from time to time to provided support on patients with complications. The Zemio team was also supported by the HIV/TB adviser, health adviser and epidemiologist from MSF headquarters. For the supply of ART, MSF ordered ARVs and OI drugs internationally and delivered them to Zemio by plane until 2015, after which supplies were partly ordered through Global Fund in Bangui. An important aspect of the program was community engagement, MSF used 50 community health workers to build acceptance of the program, report on patient outcomes and encourage patients to remain on ART.

Mortality during the study was reported directly if patients died in hospital or if the patient died in the community this was reported by community health workers who were in frequent contact with patients. As the cause of death was not always clearly reported or may have been unknown, we have considered all deaths to be HIV/AIDS related. Loss to follow-up was defined as living patients ≥6 months absent from their last appointment at the end of the study period.

We used a logistic regression model for the survival analysis, where the outcomes and time-varying covariates were aggregated by month. The probability of mortality for patient *j* in month *i* was given by a logit-transformed linear function of an intercept and covariates. Covariates were i) sex, ii) patient’s age at cohort entry in years, iii) clinical stage of OIs, iv) the number of civilian fatalities and injuries per month from armed groups, and v) abductions per month from armed groups in the patient catchment area, as recorded by the NGO Invisible Children [24]. The model intercept was permitted to vary by the number of months since the patient entered the cohort, where the monthly intercepts were drawn from a multivariate normal distribution with a covariance matrix which accounted for temporal autocorrelation between months [32].

Data cleaning and descriptive analysis was performed using R (version 3.4.4) and the models were fitted with Hamiltonian Markov Chain Monte Carlo (MCMC) using Stan (v2.17.3). We assigned vaguely informative normal prior distributions to parameters [33]. Four parallel MCMC chains were run for 40,000 iterations including burn-in, and convergence was assessed using the Gelman-Rubin statistic, the effective sample size and visual inspection [34]. We used the median of posterior parameter distributions as the measure of central tendency and the 95% credible intervals (CrI) as the measure of dispersion.

## Results

A total of 1631 HIV positive patients were recruited into the study, 1147 were female (70.3%) and the median age at first visit was 29 years (interquartile range [IQR] 22, 37). All patients were initiated on co-trimoxazole and the majority were enrolled on ART (*n* = 1491, 91.4%). In total there were 148 deaths (9.1%), of which the majority, 121 (81.8%), were among patients on ART. Patients spent a median of 27 months in the cohort (IQR 12, 48). The proportion of patients who survived the first 12 months in the cohort was 1212/1315; 0.92 (95% confidence interval [CI] 0.90, 0.93). Overall 183/1631 (11.2%) patients met the criteria for loss to follow up. Patients that were CD4 tested on their first visit (*n* = 1017) had a median count of 268 CD4 cells/μl (IQR 148, 427), this rose after 6 months on ART to a median of 403 CD4 cells/μl (IQR 240, 634; *n* = 442) and after 12 months to a median of 433 CD4 cells/μl (IQR 264, 636; *n* = 321). Patient characteristics are summarised in Table 1.

**Fig. 3:**
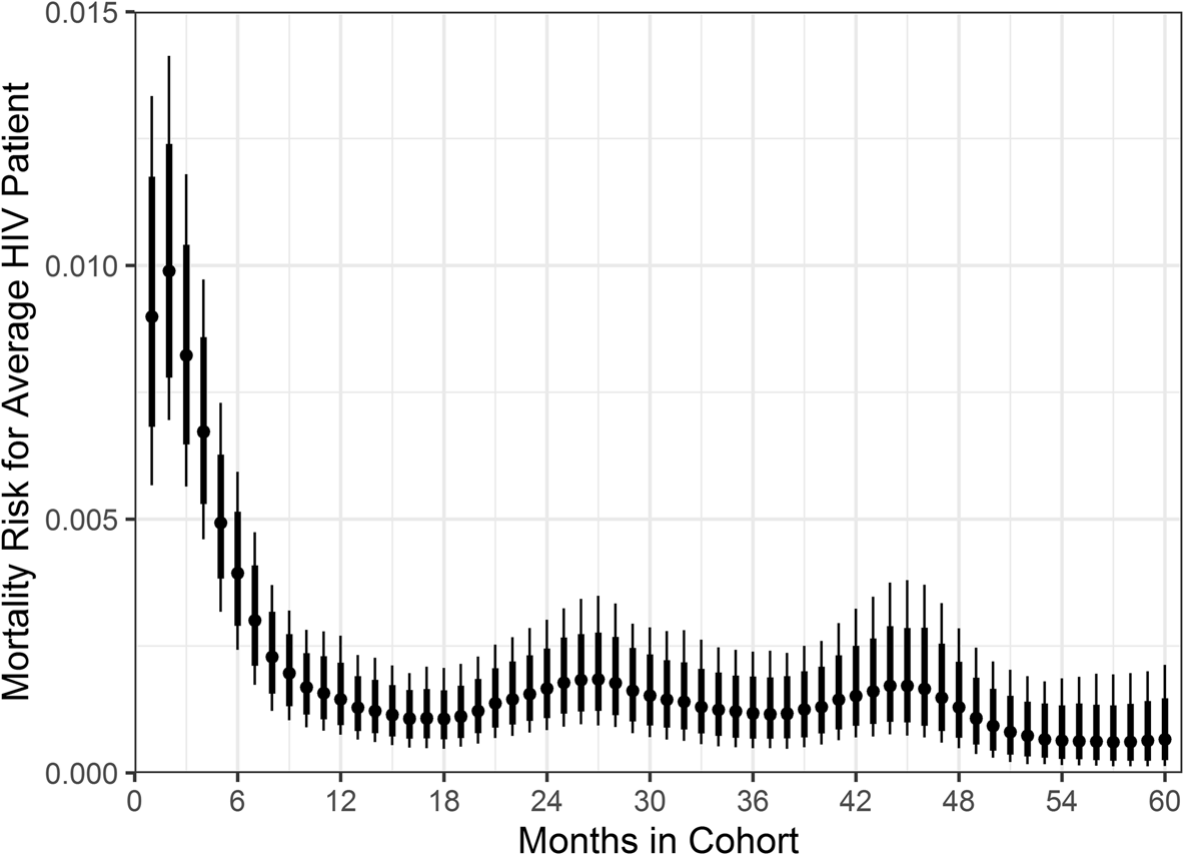
The monthly risk of mortality for an average HIV patient in the Zemio cohort. The point gives the posterior median, the broad bar the 80% posterior credible interval and the thin bar the 95% posterior credible interval. An average patient is defined as a female, 31 years old at baseline, with clinical stage 3 opportunistic infections at baseline. We also assume 2 civilian deaths or injuries per month and 21 abductions (median values for conflict explanatory variables). The risk varies by the length of time the patient is in the cohort, with the probability of mortality highest in the first 2 months, and then declines rapidly until the twelfth month. Note that these monthly probabilities are conditional on the patient having survived until that point

For the statistical analysis, we excluded three patients who had incomplete data (all three were missing both the date of enrolment and clinical staging of OIs at baseline). This therefore left 1628 patients who contributed 48,430 person-months at risk (Fig. 2). Modelling patient mortality as a series of monthly Bernoulli trials, the baseline monthly risk of mortality declined the longer the patient remained in the cohort. In the first month a patient entered the cohort, the median baseline risk (probability when all covariates are set to zero) of mortality was 9.5 × 10^− 4^ (95% CrI 4.1 × 10^− 4^, 2.1 × 10^− 3^). The risk of mortality peaked in the second month at 0.0010 (95% CrI 4.7 × 10^− 4^, 0.0023) and subsequently declined nearly seven-fold by the twelfth month to 1.5 × 10^− 4^ (95% CrI 5.8 × 10^− 5^, 3.9 × 10^− 4^). We show the risk of mortality by month in the cohort in Fig. 3, where the risk has been scaled by the covariates for an “average” patient (female, 31 years old, OI stage 3 at baseline, 2 deaths/injuries per month, 21 abductions per month); of note is the rapid decline in the monthly risk of mortality over the first year the patient is in the cohort.

The impact of covariates on the baseline risk of mortality is shown in Table 2 as odds ratios. Male sex was associated with a higher monthly risk of mortality; median odds ratio (OR) 1.7 (95% CrI 1.2, 2.3). Higher age was also associated with an increased odds of mortality; OR 1.01 (95% CrI 1.00, 1.03) for each unit (year) increase. The covariate with the highest impact on the odds of mortality was the severity of opportunistic infections (OIs) at baseline. Relative to individuals with stage 1 OIs, stage 2 OIs had an OR of 2.5 (95% CrI 2.0, 3.2), stage 3 OIs had an OR of 6.4 (95% CrI 4.1, 10) and stage 4 OIs had an OR of 16 (95% CrI 8.2, 34). The time-varying conflict covariates had a weaker association with monthly patient mortality, with the credible interval spanning unity in both instances. Fatalities and injuries from conflict had an OR of 1.00 (95% CrI 0.99, 1.01), and abductions had an OR of 1.00 (95% CrI 0.99, 1.00) on the monthly risk of mortality per unit increase.

**Table 2:** Odds ratios of covariates on the risk of monthly mortality estimated from 48,430 patient months at risk from 1628 HIV-positive patients enrolled in the Zemio HIV cohort, Central African Republic from October 2011–May 2017

Covariate (range of values)	Classification	Median odds ratio	95% Credible Interval
Patient Sex (male or female; female is baseline)	Binary	1.70	1.20, 2.33
Age at entry (0–71 years)	Continuous integer	1.01	1.00, 1.03
Clinical Staging of OIs (stage 1–4)	Continuous integer	2.52	2.01, 3.23
Civilian deaths and injuries per month (0–153)	Continuous integer	1.00	0.99, 1.01
Civilian abductions per month (0–119)	Continuous integer	1.00	0.99, 1.00

**Fig. 4:**
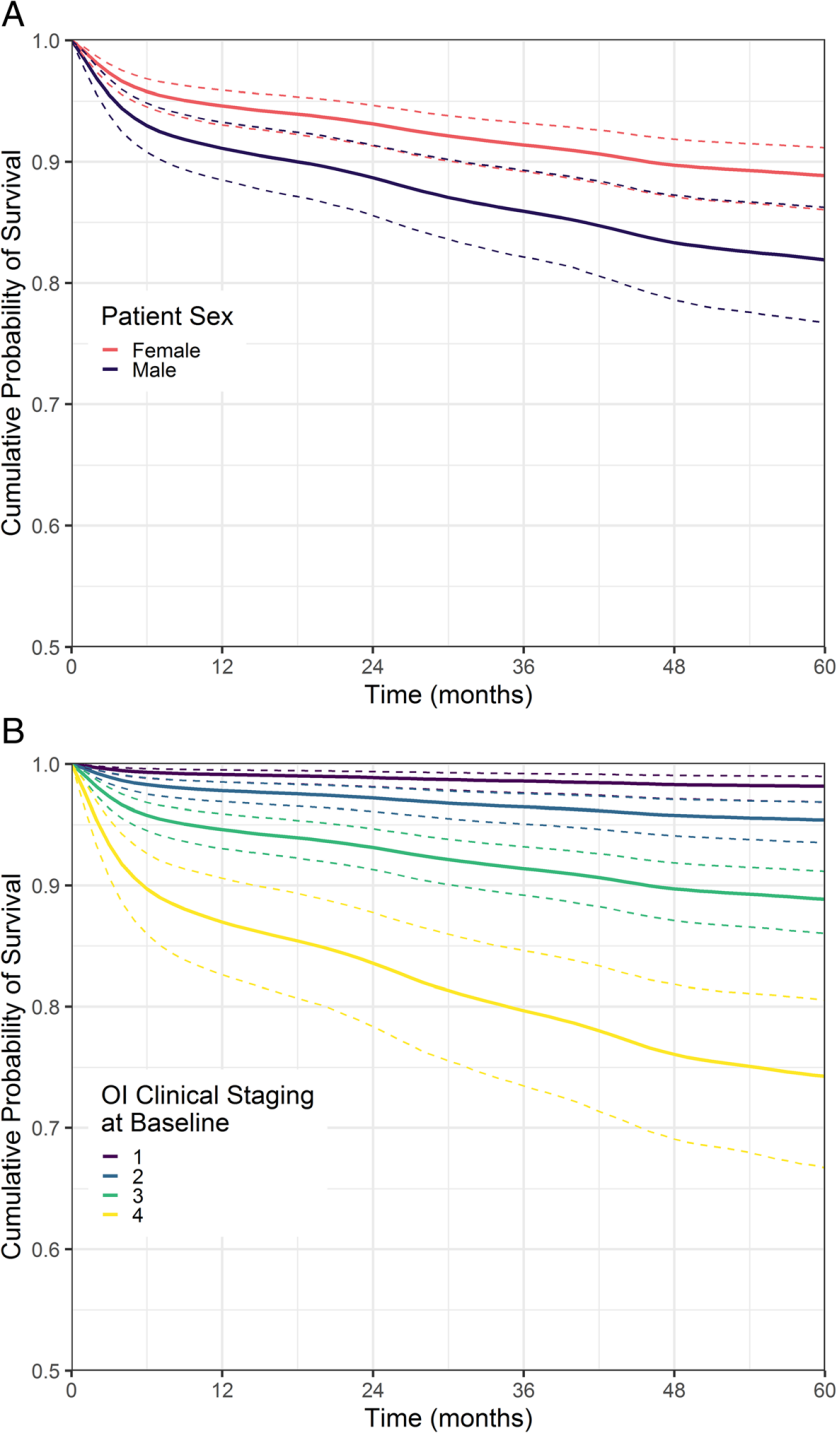
The cumulative probability of survival for HIV patients in the Zemio cohort shown **a** by sex, and **b** by clinical stage of opportunistic infections at baseline. The thick line shows the posterior median and the dotted lines show the 95% posterior credible interval. The patients stratified by gender are given covariate values of 31 years old at baseline, clinical stage 3 opportunistic infections at baseline and we assume 2 civilian deaths or injuries per month and 21 abductions for each month. The patients stratified by clinical stage of OIs, are female, 31 years old at baseline, and we assume 2 civilian deaths or injuries per month and 21 abductions for each month

The cumulative risk of mortality over the length of time spent in the cohort is shown in Fig. 4, stratified by A) patient sex and B) clinical staging (severity) of OIs at baseline. The proportion of females (31 years old, OI stage 3 at baseline, 2 deaths/injuries per month 21 abductions per months) surviving after 60 months is estimated to be 0.89 (95% CrI 0.86, 0.91) and for males with the same characteristics is 0.82 (95% CrI 0.77, 0.86). The proportion of patients (females, 31 years old, 2 deaths/injuries per month 21 abductions per months) surviving after 60 months by OI severity at baseline are 0.98 (95% CrI 0.97, 0.99), 0.95 (95% CrI 0.94, 0.97), 0.89 (95% CrI 0.86, 0.91) and 0.74 (95% CrI 0.67, 0.81) for clinical stages 1, 2, 3 and 4 respectively.

The logistic regression model showed adequate convergence according to best practice guidelines [34], with all parameters showing an effective sample size > 400 and the Gelman-Rubin statistic ≤1.01, indicating that chains had mixed well and run for sufficient iterations.

## Discussion

This study analysed the pattern of mortality among a cohort of 1628 HIV patients in Zemio, Central African Republic over 48,430 person-months at risk and determined if conflict from armed groups in the surrounding area was associated with a higher risk of patient mortality. The results from the logistic regression are strongly suggestive of no effect from i) fatalities and injuries from armed groups, or ii) abductions from armed groups on civilians on the risk of HIV patient mortality. Patient mortality was more strongly associated with both patient sex (OR 1.7 for males compared with females) and clinical staging of OIs at baseline; patients with OIs stage 4 had an OR of 16 compared with patients with stage 1 OIs, which is consistent with findings from cohort studies in low income settings [35]. The higher risk of mortality for men could be explained by poorer ART retention rates or differences in health seeking behaviour following diagnosis, which have been observed in other African settings [36–38], however we did not collect data on these confounding variables in this study.

A meta-review of mortality during ART from 18 cohort studies in West, East and Southern Africa reported survival at 12 months ranging from 0.74 to 0.92 [25], therefore our crude 12 month survival estimate of 0.92 (95% CI 0.90, 0.93) is at the higher level of this range, and comparable to estimates from conflict settings such as Bukavu, DRC, which was also estimated at 0.92 (95% CI 0.88, 0.96) [17]. This high survival rate may be partly explained by the median CD4 count we observe at baseline; 268 (IQR 148, 427; Table 1), which is higher than in other published African studies and is comparable to baseline CD4 counts observed in cohorts from developed countries (median of 234 cells/μL from European and North American cohorts) [25, 35]. Counts of CD4 cells are highly predictive of early patient mortality; patients with < 50 cells/μL were found to have a risk of mortality 2.5 times that of patients with CD4 counts ≥50 cells/μL after 12 months [25]. In our analysis we opted to use clinical staging as an explanatory variable for survival in lieu of CD4 counts as we were unable to attain results for every patient owing to ruptures in stock and the difficulty of maintaining laboratory equipment in a remote rural setting. The provision of co-trimoxazole alongside ART throughout the treatment period likely also contributed to high survival rates due to partial protection against OIs and malaria, the latter is the leading cause of death in CAR [39, 40]. Mortality was highest in the first 3 months of enrolment and peaked in the second month (Fig. 3), this is consistent with other cohort studies in SSA that showed the highest rates of mortality in the first 3 months of treatment [25, 26].

The time series of civilian fatalities, injuries and abductions by numerous armed groups shows the evolution of a long term conflict (Fig. 1c). There was a high underlying rate of civilian abductions throughout the period, predominantly by the LRA. Civilian mortality before 2017 was due mainly to attacks by the LRA or unidentified armed groups on civilians. During 2017 the region became embroiled in a larger national conflict between Christian Anti-Balaka and Muslim Ex-Seleka militias who clashed in the patient catchment area of Mboumou / Haut-Mboumou. Road blocks by the LRA, in addition to a lack of public transport made it challenging for patients to travel to Zemio for appointments or ART collection. Armed robberies of the staff compound were also a frequent occurrence and fighting inside Zemio led to expatriate MSF staff being evacuated twice over the study period [41]. Despite this instability, the pattern of mortality among HIV patients is similar to that observed in longitudinal studies in non-conflict settings, where mortality rates are largely driven by the clinical severity of OIs at baseline [42]. Furthermore, the relatively low numbers lost to follow-up (11.2% over 5 years) and the recovery of CD4-cell counts suggests that the Zemio HIV program was successful in retaining patients on ART.

As in any cohort analysis with right censored observations, our results are subject to limitations. When unreported deaths are classified as loss to follow-up this may cause the mortality rate to be biased downwards. Conversely, the mortality rate may be inflated by considering all deaths that occurred in the cohort to be HIV/AIDs related. This was a conservative decision to compensate for the lack of information on causes of death, particularly when patients died in the community rather than at the health centre. We considered the effect of conflict in the patient catchment area, measured by civilian deaths/ injuries and abductions, to be constant for all communities. Patients drawn from Obo to the east of Zemio and those drawn from the west from Rafaï are separated by 350 km, therefore a more nuanced approach would be to consider the impact of conflict at smaller spatial scales. The largest upsurge in violence against civilians also coincided with the end of the MSF program and the transition to a community led HIV program in Zemio from mid-2017 (Fig. 1c); we were therefore unable to assess the longer term impact of this interruption to treatment. Nevertheless, our study is among the first to quantitatively assess temporally varying conflict on HIV cohort mortality and the large sample size gives confidence to the parameter estimates we have obtained.

The generalisability of our findings is influenced by the representativeness of the cohort composition. Notably women far outnumber men in the Zemio cohort (70.3%). Whether HIV prevalence is higher among women in the community is unknown and depends on the pattern of transmission [43, 44]. The majority of recorded occupations among adults is agricultural workers, which is representative of the general population; The World Bank estimates that around 85% of adults in the CAR work as subsistence agriculturalists [45]. Health workers and medical staff in Zemio commented on the high level of acceptance of the HIV treatment program by the community, which was likely reinforced by the community health workers and the free healthcare offered by MSF at the health centre in a number of departments. In conflict settings where communities are less receptive to ART programs, the uptake and adherence to treatment, and subsequently the risk of mortality, may be heightened.

The Zemio HIV cohort transitioned to a community-based model of care in 2017 in order to reduce operating costs, personnel requirements, and ensure the future sustainability of the program. Community models have been shown in low-resource settings to deliver outcomes for patients that are comparable to provider-based care [46, 47]. This transition was interrupted when fighting broke out again in the town of Zemio in July 2017 (at the end of the observed period) leading MSF to temporarily suspend its operations [48]. The community model of care was continued from December 2017 onwards and MSF continues to monitor the impact of conflict on mortality and patient outcomes for HIV patients in Zemio.

## Conclusion

We have shown that the pattern of mortality among a cohort of HIV patients in the Central African Republic is driven more strongly by patient level covariates than by the evolution of the surrounding long term conflict. The levels of mortality we observed in this conflict setting are comparable to those in stable resource poor settings. We call for a renewed emphasis on increased access to ART in conflict settings.

## Data Availability

The data on aggregated monthly counts of patient mortality used in the first model and all code are available from the corresponding authors on request. Individual level patient data cannot be shared to protect patient confidentiality.
